# Neuropsychological Theory as a Basis for Clinical Translation of Animal Models of Neuropsychiatric Disorder

**DOI:** 10.3389/fnbeh.2022.877633

**Published:** 2022-05-10

**Authors:** Neil McNaughton

**Affiliations:** Department of Psychology and Brain Health Research Centre, University of Otago, Dunedin, New Zealand

**Keywords:** animal model, anxiety, disorder, clinical translation, neuropsychology, theory, biomarker

## Introduction

Neuropsychiatric disorders are poorly defined. The DSM-5 has many hundred categorical disorders (American Psychiatric Association, 2013) as does the only partially matching ICD-10 (World Health Organization, 1992). But the categories are based on symptoms (and often a required duration), not neurobiological causes, which are likely dimensional and reflected in traits (Kotov et al., 2021; Michelini et al., 2021; DeYoung et al., 2022). There can also be problems with labeling. For example, are fear and anxiety synonyms, metonyms, or antonyms (McNaughton, 2018)? With this confusion, it is no surprise that, even with broad-spectrum pharmaceuticals and well-developed psychological therapies, 30–60% of those receiving first-line treatment have continuing impairment; and the criteria for multi-treatment resistance are not well defined (Bokma et al., 2019).

These patient-level problems make it hard to construct good, matching, animal models (McNaughton and Zangrossi, 2008). Indeed, I will argue that it should be the more successful animal models that provide the basis for proper clinical diagnoses. Many models have been constructed for face validity; but have poor prediction of therapeutic drug action [e.g., the elevated plus maze does not reliably detect serotonergic drugs (Handley et al., 1993; Griebel, 1995) and a wide range of tests—e.g. holeboard, Geller-Seifter, social interaction—each has its own profile of detection (Cryan and Sweeney, 2011)] and, being based on behavior in healthy animals, no clear relationship to clinical disorder—given this discrepancy it is amazing that any animal models are predictive at all. The capacity of such animal models to predict effects on clinical disorder, is one among many reasons for taking a trait perspective—as does the anxiolytic effect in healthy people of the GABA_A_ agonist, ethanol. “Psychiatry has proven to be among the least penetrable clinical disciplines for productively marrying knowledge of human pathology with animal behavior to develop satisfactory *in vivo* animal models for evaluating novel treatment approaches” (Cryan and Sweeney, 2011).

I argue that the answer lies in using strong neuropsychological theory as a basis for model construction, translation, and understanding of psychiatry disorders. As we noted elsewhere:

“Theory influences what we mean by the word “anxiety”, what we require of any animal model, and what specific theoretical constructs are embedded in any specific animal model of anxiety. We argue that, in the ideal case, the animal models we use should be embedded in a large-scale theory that integrates all of the theoretical levels of each animal model. We argue that face validity of a model should be ignored and that true predictive validity reduces ultimately to construct validity. So all models should aim to have construct validity based on strong theory. Theoretical analysis shows that anxiety should be distinguished from fear; that different anxiety disorders should be distinguished from each other; and that the components of any single apparent type of anxiety can have distinct neural control. Theory can show how a model is unsatisfactory, but it can also show that it is not the model but rather our translation from the clinical situation that is faulty. To model the many flavors of clinical disorder and variations in drug effectiveness, we must use theory to link multiple animal models, neural analysis and pharmacological analysis. The goal is to provide us with truly predictive tests that can be used for drug discovery as well as drug development. Most importantly, theory is required if we are to correctly match a particular measure from a particular model with the clinical entity we desire to model” (McNaughton and Zangrossi, 2008).

## Generating Strong Neuropsychological Theory

Over 50 years ago, Gray (1970), compared the effects of the anxiolytic barbiturate sodium amylobarbitone, septal lesions, and hippocampal lesions; and proposed on the basis of similarity across only a few behavioral experiments that the septo-hippocampal system is a key site for anxiolytic (as opposed to other) drug actions. He also proposed that impairment of the theta rhythm that is seen in the hippocampus and controlled by the septum was the key to these common effects. Note that this key hypothesis was based on drugs not psychology, with the specific nature of the similarly-affected behaviors something that needed considerable work to determine.

To develop a full theory from this hypothesis, the neural basis of anxiolytic action, and the psychological nature of the drugs' effects, then received 30 years of progressive development (Gray, 1982; Gray and McNaughton, 2000; McNaughton and Gray, 2000) that retained the underlying hypothetical bedrock while elaborating on the superstructure. Importantly, despite the appearance of completely new classes of drugs (benzodiazepines^1^, buspirone—a serotonin_1A_ agonist, specific serotonin reuptake inhibitors, pregabalin—a calcium channel agent, and ketamine, the anxiolytic mechanism of which is unknown), the positive and negative predictions of the theory remained intact. For example, both benzodiazepines and buspirone impair control of hippocampal theta rhythm (Zhu and McNaughton, 1991, 1995a), impair hippocampus-sensitive learning in the Morris water maze (McNaughton and Morris, 1987, 1992), and impair hippocampus-sensitive behavioral inhibition (Gray and McNaughton, 1983; Zhu and McNaughton, 1995b). We have proved that the benzodiazepines affect behavioral inhibition via an action on one of the locations that controls theta rhythm (Woodnorth and McNaughton, 2002) and that theta rhythmicity, in and of itself, is important for spatial learning (McNaughton et al., 2006; Ruan et al., 2011).

This may seem a long road to have traveled but, as we will see, it leads to more than one desirable destination. A good theory, properly applied, solves many problems—and, as Newton showed, need not be complicated in its core elements even though their working out can be another matter (as shown by the 3-body problem).

## From Theory to Construct Validity

It is important to note that the anxiety-septohippocampal-theta theory implies construct validity for some models and not others. Operant tests of “behavioral inhibition” have long had anxiolytic predictive validity; and fell out of favor because of their cost. But the role of goal conflict in the theory (Gray and McNaughton, 2000) gives the bulk of them stronger construct validity; and also explains the lack of effect of anxiolytics on *action* inhibition (McNaughton et al., 2013; Shadli et al., 2015), which is functionally distinct but lexically confusable. Similarly, the theory provides a good theoretical basis for contextual conditioning (Luyten et al., 2011), and the elevated T-maze (McNaughton and Zangrossi, 2008) and but less so the elevated plus maze (Pellow et al., 1987) unless ethological measures are used (Cole and Rodgers, 1994, 1995; Rodgers and Cole, 1994).

Here it is worth noting that the only current model of clinical anxiolytic action that has no false positives nor false negatives (McNaughton et al., 2007) in 40 years of testing (McNaughton and Sedgwick, 1978) is reticular stimulation elicited hippocampal theta rhythm. This is, of course, one of the key foundational elements of the theory. But it has also withstood the challenge of the progressive appearance of new classes of anxiolytic and of recent predictive tests (Engin et al., 2009; Siok et al., 2009). Importantly for its construct validity, specific manipulations of the theta control system alter behavior in a manner consistent with the psychological aspects of the theory when they alter theta in a fashion consistent with theta changes being the basis for their behavioral actions.

## From Model to Translational Test

It might seem difficult to take a rat model that uses depth stimulation and recording in rats and use it to develop an equivalent human test. This is where theory can provide a bridge.

In the theory (Gray and McNaughton, 2000) hippocampal theta is necessary but not sufficient for goal conflict processing and will be present even when the hippocampus has no functional output. The prefrontal cortex can show its own forms of theta (Mitchell et al., 2008) but becomes synchronous with hippocampal theta during, e.g., risk assessment behavior (Young and McNaughton, 2009) and novelty detection (Park et al., 2021); and theta (and other rhythms) changes across the hippocampus-amygdala-prefrontal network in response to stress (Merino et al., 2021). This suggests that we should be able to use prefrontal scalp EEG to record theta rhythmicity that is functionally equivalent to the hippocampal theta in the rat model.

The psychological core of the theory is goal conflict (Gray and McNaughton, 2000). We therefore tested for frontal theta rhythmicity linked to goal conflict in a simple gain/loss-based approach/avoidance task in student participants (Neo and McNaughton, 2011; Neo et al., 2020) and found a conflict-related power increase at the right-frontal site, F8. Right frontal cortex, and the right inferior frontal gyrus in particular, are involved in stopping (a key output of the goal conflict system) in the stop signal task (SST). So, we used the SST and found that it generated a goal-conflict specific “theta” rhythmicity (GCSR) at F8 (Neo et al., 2011).

The SST has the advantage, for clinical work, that it does not use monetary gain and loss. We, therefore, proceeded to validate GCSR within the theory by showing that it is sensitive to the three main types of specifically anxiolytic (i.e. not antipanic) drugs (McNaughton et al., 2013), similarly validated an improved version of the task (Shadli et al., 2015, 2020), demonstrated its relation to handedness (Shadli et al., 2021a), and demonstrated that its value is high in a subgroup of those with high trait anxiety scores and DSM “anxiety disorder” diagnoses (Shadli et al., 2021b).

## Discussion

It is tempting to ask, at this point, “What sort of DSM anxiety does GCSR represent?”. However, as a biomarker for a causal agent (equivalent to SARS-Cov-2), it will be linked to a wide range of symptomatic expressions of its disorder (equivalent to COVID-19) and show both positive and negative discrepancies from any DSM symptom-based class (equivalent to “flu-like respiratory infection”). The key original problem is that current diagnoses do not map to the underlying biological disorder that generates the symptoms.

**Figure 1:**
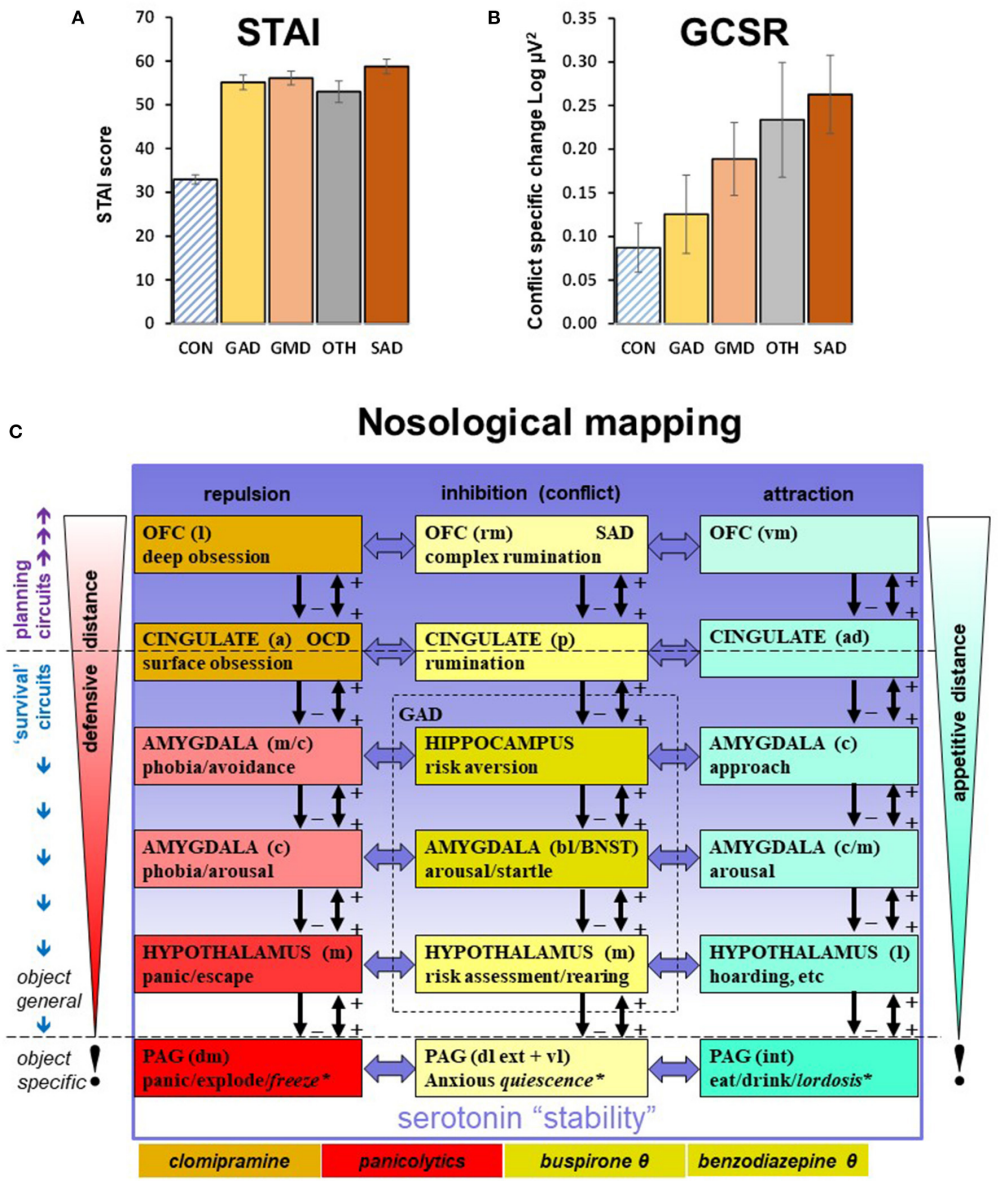
**(A)** The relationship of Spielberger State Trait Anxiety Inventory (Spielberger et al., 1983) trait values (STAI) to DSM (American Psychiatric Association, 2013) diagnosis in the same groups as B. Bars are ±SEM. **(B)** The relationship of goal conflict specific rhythmicity (GCSR, 4–7 Hz maximum value) to DSM diagnosis. The data are from (Shadli et al., 2021b) with permission of the author. Note that the trait anxiety scores are high (clinical cases are usually >45), and very similar across the groups. GCSR appears elevated across all diagnoses but varies across the groups (there is no obvious factor controlling this variation). CON, community control; GAD, generalized anxiety disorder; GMD, GAD + major depression; SAD, social anxiety disorder; OTH, mixed other anxiety-related diagnoses with small N per diagnosis. **(C)** Nosological mapping to hierarchical systems. Goal attraction, goal repulsion, and goal inhibition (activated by conflict between goals) are each controlled by systems in which modules are organized hierarchically in relation to motivational distance (from contacting to distant) and neural location (caudal to rostral). Conservation of modulatory control during phylogeny (McNaughton, 2020) means that hormonal compounds, e.g., benzodiazepine receptor ligands, and neuromodulators, e.g., serotonin, can target all the modules of a specific system (as with benzodiazepines and goal inhibition; yellow highlight) or all the modules of several systems (as with serotonin). Note that in the case of serotonin (most obviously via specific serotonin reuptake inhibitors), its effects (indicated by the gradation of the purple shading) appear to be to shift control from lower to higher levels of the systems (Carver et al., 2008) rather than to increase or decrease activity across an entire system. There is also the capacity for more localized dysfunction and pharmacological specificity, as with obsession (orange highlight) and panic (red highlight). Figure and legend based on (McNaughton et al., 2016; Silva and McNaughton, 2019; McNaughton, 2020) with permission of the author. a, anterior; b, basal; c, central; d, dorsal; ext, external; int, internal; l, lateral; m, medial; p, posterior v, ventral; BNST, bed nucleus of the stria terminalis; SAD, Social Anxiety Disorder; OCD, obsessive compulsive disorder; OFC, orbitofrontal cortex; PAG, periaqueductal gray; θ, these compounds affect the system as a whole by reducing theta rhythmic input.

Figures 1A,B show that, despite very similar scores on the trait scale of the Spielberger State Trait Anxiety Inventory (Spielberger et al., 1983), the diagnostic groups differ in a non-categorical way in terms of GCSR. This fits both previous doubts about the current categories and the recent move to a trait perspective on psychopathology (Kotov et al., 2021; Michelini et al., 2021; DeYoung et al., 2022). It also fits the nosological mapping of the neuropsychological theory (Gray and McNaughton, 2000; McNaughton and Corr, 2004; McNaughton, 2020) summarized in Figure 1C. The first important feature of this nosology is that theta is a modulator across the range of conflict control structures, with its strongest effects in the middle of the hierarchy (see shading in figure). Thus, the disorder for which GCSR is a biomarker will represent only one trait component of the possible conflict-related disorders. The second important feature is that within the separate repulsion system, there are distinct areas controlling obsession (and so linked to obsessive compulsive disorder) and panic (and so linked to panic disorder); each with their own pharmacological sensitivities and so capacity for distinct contributions to disorder; while, conversely, serotonergic modulation can impact on all systems; as can noradrenergic modulation, which is also involved in anxiety control but to a more limited extent (Gray, 1982; Gray and McNaughton, 2000; McNaughton and Gray, 2000). These are all immediate targets for the search for relevant models that will deliver new biomarkers. A third particularly important feature is that this neuropsychology implies only a loose connection between causes of disorder and symptoms. Pathological panic can generate anxiety in an otherwise normal anxiety system, and vice versa, with the capacity for a vicious cycle (McNaughton and Corr, 2016); while a dysfunctional serotonin system could generate a combination of pathological panic and pathological anxiety.

On this view, problems with previous animal models of psychiatric disorder may have resulted from both inappropriate assumptions behind perception of face validity and inappropriate relation of symptoms to diagnostic categories. Strong theory that accounts for the fundamental similarities of species, while allowing for their species-specific superficial expression should provide a way forward.

## Author Contributions

The author confirms being the sole contributor of this work and has approved it for publication.

## Conflict of Interest

The author declares that the research was conducted in the absence of any commercial or financial relationships that could be construed as a potential conflict of interest.

## Publisher's Note

All claims expressed in this article are solely those of the authors and do not necessarily represent those of their affiliated organizations, or those of the publisher, the editors and the reviewers. Any product that may be evaluated in this article, or claim that may be made by its manufacturer, is not guaranteed or endorsed by the publisher.

## Footnotes

^1^The benzodiazepines act on the same GABA_A_ receptor as do the barbiturates, meprobamate, and other classical anxiolytics—but they do so by changing the way the GABA site works (Haefely, 1990), not through a site that acts directly to open the channel. This was a breakthrough for toxicity and related side effects.
